# Caveolin-1 in vascular health and glaucoma: A critical vascular regulator and potential therapeutic target

**DOI:** 10.3389/fmed.2023.1087123

**Published:** 2023-01-24

**Authors:** Jing Hong Loo, Zhaoran Wang, Rachel S. Chong

**Affiliations:** ^1^Yong Loo Lin School of Medicine, National University of Singapore, Singapore, Singapore; ^2^Duke-NUS Medical School, Singapore, Singapore; ^3^Glaucoma Department, Singapore National Eye Center, Singapore, Singapore; ^4^Ocular Imaging Department, Singapore Eye Research Institute, Singapore, Singapore

**Keywords:** glaucoma, Caveolin-1 (Cav-1), systemic vascular risk factors, ocular blood flow, neurovascular coupling (NVC), glaucoma therapy

## Abstract

Caveolin-1 (Cav-1) is an integral scaffolding membrane protein found in most cell types. Cav-1 has been found to contribute significantly to ocular function, with mutations of Cav-1 being associated with a genetic risk of glaucoma development. Raised intraocular pressure (IOP) is a major modifiable risk factor for glaucoma. Cav-1 may be involved in both IOP-dependent and independent mechanisms involving vascular dysregulation. Systemic vascular diseases including hypertension, diabetes and hyperlipidaemia, have been shown to be associated with glaucoma development. Cav-1 is closely interlinked with endothelial nitric oxide synthase pathways that mediate vascular function and prevent cardiovascular diseases. Endothelial nitric oxide synthase and endothelin-1 are key vasoactive molecules expressed in retinal blood vessels that function to autoregulate ocular blood flow (OBF). Disruptions in the homeostasis of OBF have led to a growing concept of impaired neurovascular coupling in glaucoma. The imbalance between perfusion and neuronal stimulation arising from Cav-1 depletion may result in relative ischemia of the optic nerve head and glaucomatous injury. OBF is also governed by circadian variation in IOP and systemic blood pressure (BP). Cav-1 has been shown to influence central BP variability and other circadian rhythms such as the diurnal phagolysosomal digestion of photoreceptor fragments and toxic substrates to maintain ocular health. Overall, the vast implications of Cav-1 on various ocular mechanisms leading to glaucoma suggest a potential for new therapeutics to enhance Cav-1 expression, which has seen success in other neurodegenerative diseases.

## Caveolin-1 in vascular health

Caveolin-1 (Cav-1) is a major coat protein of caveolae, which are flask-shaped invaginations of the plasma membrane ubiquitously found in various cell types, particularly adipocytes, endothelial cells, epithelial cells and fibroblasts. Caveolae have been discovered to play roles in lipid transportation (1), membrane traffic (2), and signal transduction (3). Although there are three caveolin genes identified in mammals, namely Cav-1, -2, and -3, Cav-1 is notably essential for caveolae formation and function (4, 5). Through a multitude of signaling cascades, Cav-1 has been implicated in cardiovascular disease, atherosclerosis, diabetes, cancer, and a variety of degenerative muscular dystrophies (6). In the cardiovascular system, Cav-1 particularly contributes to the functions of endothelial cells *via* interacting with endothelial nitric oxide synthase (eNOS) and regulating the release of nitric oxide (NO) (7).

## Caveolin-1 involvement in glaucoma

While Cav-1 has been extensively studied in extra-ocular diseases, its role in ocular function and diseases has only recently received attention. Cav-1 is expressed abundantly in Muller glia, retinal and choroidal vasculature, and retinal pigment epithelium (RPE) (8). Mutations of Cav-1 gene are associated with an increased genetic risk of primary open-angle glaucoma development across various population cohorts (9–12).

Glaucoma is a neurodegenerative disease characterized by progressive loss of retinal ganglion cells (RGC) and optic nerve degeneration that results in irreversible visual field deficits. Raised intraocular pressure (IOP) is a major modifiable risk factor for glaucoma (13). The effect of Cav-1 deficiency on IOP homeostasis has been evaluated in various pre-clinical studies, with Cav-1 deficient mice displaying significantly higher IOP (14–16). A postulated mechanism underlying ocular hypertension in Cav-1 deficiency is the resultant overreactive eNOS signaling pathways. While NO is a potent vasodilator and has a crucial role in lowering IOP, chronic dysregulation of eNOS may lead to outflow tract dysfunction. Indeed, Cav-1 depletion has been independently associated with increased conventional outflow resistance leading to decreased drainage of aqueous humor from the anterior chamber (17). Additionally, new findings have shed light on Cav-1 potential role as mechanosensors in the Schlemm's canal and trabecular meshwork that protects against mechanical stress from IOP fluctuations (18). Cav-1 may contribute to increased IOP and increase the susceptibility of the optic nerve head (ONH) to cellular damage due to altered outflow tract mechanoprotection.

Cav-1 has also been implicated in altering vascular function, both systemically and within the eye. These alterations in vascular profile may contribute to greater glaucoma risk, as described in this mini-review.

## The role of Cav-1 in mediating systemic vascular risk factors for glaucoma

Various systemic vascular risk factors including hypertension or hypotension, diabetes mellitus, hyperlipidaemia, atherosclerotic diseases and migraine have been associated with glaucoma development. Hypertension has a direct causative link to glaucoma risk by means of increased ciliary blood flow and aqueous humor production coupled with decrease outflow due to elevated episcleral venous pressure (19). Hypotension is particularly associated with normal-tension glaucoma since it lowers ocular perfusion pressure (OPP), resulting in optic nerve ischemia and glaucomatous degeneration (20). Circadian variation of blood pressure (BP) may have a role in glaucoma development too. A meta-analysis in 2015 pooled evidence from epidemiological studies and established nocturnal BP fall as a risk factor for progressive visual field losses in glaucoma (21). In presence of the other vascular risk factors, nocturnal dipping exacerbates poor optic nerve perfusion and glaucomatous optic neuropathy (22). The association between diabetes and glaucoma may be explained by a few key mechanisms. Hyperglycaemia and dysregulation in lipid metabolism results in oxidative stress, vascular dysregulation and eventual neuronal injury (23–25). Hyperglycaemia of the aqueous humor also leads to structural remodeling at the trabecular meshwork and impaired aqueous humor outflow (26). Atherosclerotic diseases include a spectrum of disease conditions from coronary artery disease to peripheral vascular disease and stroke. The association between atherosclerotic diseases and glaucoma has been extensively studied (27–30), however current evidence is insufficient to support a direct causal relationship between the two due to potential confounding factors from the underlying pathophysiological processes involved. Finally, migraine is associated with systemic vasospasm causing relative ischemia, thereby increasing the risk of glaucoma, particularly normal-tension glaucoma (31–33).

Many of the aforementioned systemic vascular risk factors have been linked to Cav-1. From diabetes to lipid disorders and pulmonary fibrosis, Cav-1 plays an integral role in maintaining vascular homeostasis and controlling atherosclerosis formation through lipoprotein trafficking across the vascular endothelium (34–36). Central to this physiology is the regulatory setup of Cav-1/eNOS. eNOS is constitutively expressed in vascular endothelium and produces the vasodilatory gas NO which maintains endothelial function and health (37). eNOS bounded to caveolae is rendered inactive by its direct association with caveolin scaffolding domain of Cav-1 (38). Cav-1 directly competes with calmodulin (an activator of eNOS) for binding to the active site of eNOS (39). Furthermore, Cav-1 also regulates eNOS expression levels by inhibiting serine/threonine amino acid kinase Akt phosphorylation of eNOS, thus governing the basal level of NO in endothelial cells (40). While Cav-1 depletion is characterized by chronic hyperactivation of eNOS, a decoupling of the de-inhibited eNOS may occur, thus resulting in a decreased bioavailability of NO (41). Reduced NO production is associated with vascular dysfunction and cardiovascular mortality (42).

## Cav-1 mediated regulation of ocular blood flow *via* NO-dependent and independent pathways

Autoregulation of ocular blood flow (OBF) in the retinal vasculature enables a relatively stable supply of blood and metabolites despite fluctuation in OPP (43). OPP is calculated as derived from the subtraction of IOP from mean arterial pressure (44). Variations in mean arterial pressure and IOP results in corresponding variations in OPP. Within a range of OPP, OBF remains constant due to autoregulation of vascular tone in the retinal and ONH (45).

Two vasoactive factors, namely NO and ET-1, are crucial in the autoregulatory mechanism (46). NO is a potent vasodilator released by endothelial cells and acts on pericytes to cause vasodilation (47). ET-1 is a potent vasoconstrictor that exerts its effect *via* ETA, ETB1, and ETB2 receptors. ETA and ETB2 receptors are found on vascular smooth muscle cells and causes vasoconstriction while ETB1 is found on endothelial cells and cause vasodilation (48). The counterregulatory effects of NO and ET-1 maintains an appropriate vascular tone and constant blood flow to the ONH.

Impaired autoregulation is seen in glaucomatous optic neuropathy. This arises from cellular dysfunction leading to an imbalance of vasoactive factors and ischemia at the ONH (49, 50). Numerous studies have shown that elevated levels of ET-1 are associated with disease pathology (51–54). Blocking of ET-1 receptors in mice increased OBF and protects from glaucomatous injury (55). Alterations in NO signaling pathways either through upregulation or downregulation are also implicated in glaucoma. High NO may increase ocular perfusion but cause oxidative stress and injury to neurons due to formation of reactive oxygen species (56). Decreased NO levels are found in the aqueous humor of glaucoma patients (57). Characteristic RGC loss and vascular dysfunction seen in glaucoma are more prominent with decreased NO production and impairment in its downstream NO-cGMP signaling pathways (58).

ET-1 and NO dysregulation is partly mediated by Cav-1. The effect of Cav-1 on NO homeostasis has been explained in the previous section. An intrinsic regulatory interaction also exists between Cav-1 and ET-1. Both the scaffolding domain and C-terminal domain of Cav-1 can bind to ET receptors and this localizes the complex to the caveolae membrane (59); it has been suggested that compartmentalization of ETB receptor/Cav-1 complexes within caveolae ensures signal transduction and prevents rapid endocytosis of the receptor (60). An early study demonstrated that disruption of caveolae structure significantly diminishes ET-1-induced phosphorylation of ERK 1/2 and subsequent signal propagation (60). This interplay between Cav-1 and mediators of vascular tone suggests the crucial role of Cav-1 in ocular vascular health. Cav-1 deficiency is associated with vascular dysregulation which may predispose to structural neuronal injury at the ONH in the presence of existing stressors like IOP, thus exacerbating disease progression (61).

Cav-1 depletion is also associated with disruptions in blood-retinal barrier integrity and venous morphology, that are independent of eNOS activity. Gu et al. have reported hyperpermeability of the large branch retinal veins of the superficial retina, and enlargement of retinal veins in Cav-1 knockout mice (62). These alterations were found to be independent of NOS-expression and activity. It is therefore possible that Cav-1 mediates vascular dysfunction in the eye through both NO-dependent and independent mechanisms, where the former regulates capillary dilation and the latter stabilizes vessel wall integrity in retinal veins.

## Neurovascular coupling in glaucoma

Neuronal activity is tightly matched to OBF in the eye in what is termed as neurovascular coupling (NVC) (45). An increase in neuronal stimulation is associated with a corresponding increase in blood flow to meet the metabolic requirements of the retinal tissue. This NVC response is mediated by the neurovascular unit which comprises vascular cells, glial cells and neurons (63, 64). Defective NVC has been described in primary open-angle glaucoma (65, 66). In response to flicker-light stimulation, the increase in OBF in glaucoma patients was found to be significantly lower than that of healthy subjects (67). Various mechanisms may explain the defective NVC response in glaucoma. Firstly, glaucoma is characterized by RGC apoptosis due to various possible causes involving raised IOP, oxidative stress and mitochondrial dysfunction—decreased neuronal signaling from RGCs may drive reduced NVC (68–70). Secondly, decreased gap junction expression in the retinal and ONH also affects communication between cells of the neurovascular unit (71, 72). Lastly, the integrity of retinal barrier is compromised due to the loss of tight junctions (73), leading to both compromised blood supply and transendothelial migration of inflammatory cells causing further neuronal injury (74).

On the back of increasing evidence of Cav-1 involvement in glaucoma, our own study in Cav-1 knockout mice showed defective NVC at the ONH as assessed by laser speckle flowgraphy (16). This is associated with changes in vessel morphology as well as a decrease in electrophysiological function of RGCs (16). While the temporal association has yet to be clearly-established, it is possible that vascular dysfunction contributes to defective NVC which is associated with early functional RGC injury before structural losses are seen. Apart from its role in mediating vascular tone as described in the previous sections, Cav-1 may influence microvascular structural characteristics by downregulating vascular endothelial growth factor (75). Defective Cav-1 promotes angiogenesis, but the excessive vascular branching pattern may lead to poorer perfusion instead (76). These findings support the theory that defective Cav-1 is associated with vascular dysfunction and impaired NVC. However, the precise involvement of glial cells or retinal microvasculature in regulating the NVC process remains to be seen—particularly in the context of glaucoma.

## Cav-1 and disruption of circadian rhythms

Numerous studies have shown that circadian variation in BP, IOP, and OPP are risk factors for the development of glaucoma. Progression of visual field loss in glaucoma patients has been associated with a larger range of diurnal IOP fluctuations and nocturnal pressure spikes (77–79). IOP tends to be higher at night due to decreased aqueous humor drainage *via* the trabecular meshwork and uveoscleral pathway (80). Similarly, diurnal variation in BP and nocturnal dipping may contribute to glaucoma pathogenesis as well (81, 82). Nocturnal BP reduction is attributed to a fall in sympathetic tone with reduced circulating levels of catecholamines (83). OPP is driven by a complex interplay between BP and IOP; fluctuations in either will translate to variations in OPP (84). Abrupt variations in OPP beyond the capacity of autoregulatory mechanisms may thus cause unstable OBF (85, 86), triggering a sequence of ischemic and reperfusion injury at the ONH.

Limited studies have described Cav-1 involvement in circadian rhythms disruptions causing glaucoma development. An experimental study by Desjardins et al. (87) showed that Cav-1 deficient mice exhibit decreased very low frequency BP variability. Administration of caveolin scaffolding domain reversed this drop in BP variability. The authors attributed this to the increased NO production *ex vivo* arising from reduced allosteric inhibition by Cav-1. While the bandwidth of spectral analysis cannot be directly applicable to human, the study does provide invaluable insights regarding the function of Cav-1 on NO production and control of central BP variability. Another circadian rhythm implicated in Cav-1 depletion is the diurnal pattern of renewal of photoreceptor outer segment (88). RPE supports photoreceptors neurons *via* the diurnal clearance of outer segment fragments (89). Cav-1 depletion impairs phagolysosome degradation by reversing the diurnal activity of enzymes in the RPE (88). Rod photoreceptor visual function is found be decreased with Cav-1 knockout (8).

While the present evidence for cav-1 involvement in circadian regulation remains scant, the unique role of Cav-1 in mediating ocular perfusion *via* multiple pathways warrants further studies into how circadian disruptions may influence Cav-1 function.

## Potential therapeutic targets

Current treatment for glaucoma relies heavily on ocular hypotensive medications. Reduction in IOP has proven effective in preventing and slowing disease progression (85). In addition to its effectiveness, IOP-lowering medications exhibit minimal systemic adverse effects and high rates of patient tolerability (90). However, continued disease progression occurs in a small subset of patients despite adequate IOP lowering (91, 92). Furthermore, a modest proportion of patient experienced glaucomatous optic neuropathy despite having normal IOP, in what is termed as normal-tension glaucoma (93). This suggests that there are other IOP-independent mechanisms that may contribute to glaucoma development (94).

The multiple roles of Cav-1 in modulating ocular health and glaucoma risk suggest the potential for new therapeutic strategies that increase Cav-1 expression or augment its downstream signaling. While research on Cav-1 therapeutics remains in its infancy, success with Cav-1 gene therapy for chronic diseases have been described in few recent studies. Lin et al. demonstrated that electroporation-mediated transfer of the Cav-1 gene protects against bleomycin-induced pulmonary fibrosis in mouse lungs *via* downregulation of inflammasome activity and reduction in monocyte recruitment and circulating cytokines (95). The use of electroporation to deliver gene targets is currently being explored in clinical trials for cancer and vaccines (96). It thus remains to be seen if the promising outcomes of Cav-1 gene therapy for idiopathic pulmonary fibrosis can be replicated in humans as well. Cav-1 therapy has also been shown to preserve or delay neurodegeneration in a preclinical model of Alzheimer's disease. Wang et al. showed that synapsin-promoted Cav-1 gene therapy was able to maintain neuronal and synaptic morphology and preserve hippocampal function such as memory and learning in mice with Alzheimer's disease (97). Further translational or clinical research may focus on whether the therapeutic potential of Cav-1 can be exploited for neuroprotective effects in the human eye, possibly averting RGC loss and glaucoma development.

## Conclusions

Raised IOP has long been regarded as the only modifiable risk factor for glaucoma. However, adequate IOP lowering with anti-glaucoma medications may not always deter glaucoma progression. Hence, other factors independent of IOP may be involved in the complex pathogenesis of glaucoma. The “vascular theory” affecting neuronal function has gained attention recently with new evidence showing vascular dysregulation may precede RGC loss (98). Patients with cardiovascular risk factors are at an increased risk of glaucoma. Dysregulation of OBF due to altered levels of vasoactive substances may lead to disruption in blood supply of the ONH. Impaired NVC can also cause a mismatch of neuronal stimulation and ocular perfusion. All these disturbances in vascular function may manifest as altered vessel morphology and vascular dropout seen in early glaucoma (99).

Cav-1 plays an important role in regulating various pathways involved in the “vascular theory” of glaucoma. There is consistent evidence describing the association between Cav-1 depletion and systemic cardiovascular disease, impaired autoregulation and defective NVC. While the underlying mechanism has not been fully elucidated, understanding this crucial association may pave the way for future therapeutics that focus on restoring vascular health to avert glaucomatous degeneration. Cav-1 therapeutics have shown promising outcomes for other disease, raising hopes that a similar approach can be applied to glaucoma prevention. Future research should focus on exploring the intricate interplay between Cav-1 and vascular dysregulation and exploiting the translative potential of Cav-1 therapy for alternative glaucoma treatment.

## Author contributions

JHL wrote the first draft of the manuscript. ZW formatted and proofread the manuscript. RC provided overall leadership and wrote sections of the manuscript. All authors contributed to manuscript revision, read, and approved the submitted version.
